# *Streptococcus* *equi* subsp. *zooepidemicus* Infections Associated with Guinea Pigs

**DOI:** 10.3201/eid2101.140640

**Published:** 2015-01

**Authors:** Karen Gruszynski, Andrea Young, Seth J. Levine, Joseph P. Garvin, Susan Brown, Lauren Turner, Angela Fritzinger, Robert E. Gertz, Julia M. Murphy, Marshall Vogt, Bernard Beall

**Affiliations:** Virginia Department of Health, Richmond, Virginia, USA (K. Gruszynski, S.J. Levine, J.M. Murphy);; Virginia Department of Health, Manassas, VA (A. Young);; Virginia Department of Agriculture and Consumer Services, Richmond (J.P. Garvin, S. Brown);; Department of General Services, Richmond (L. Turner, A. Fritzinger);; Centers for Disease Control and Prevention, Atlanta, GA, USA (R.E. Gertz, Jr., B. Beall);; Virginia Department of Health, Chesterfield, VA (M. Vogt);; Chippenham Johnston-Willis Medical Center, Richmond (M. Vogt)

**Keywords:** *Streptococcus equi* subspecies *zooepidemicus*, zoonoses, bacteria, guinea pigs, necrotizing fasciitis, PFGE, MLST, Virginia, United States

## Abstract

*Streptococcus equi* subsp. *zooepidemicus* is a known zoonotic pathogen. In this public health investigation conducted in Virginia, USA, in 2013, we identified a probable family cluster of *S*. *zooepidemicus* cases linked epidemiologically and genetically to infected guinea pigs. *S*. *zooepidemicus* infections should be considered in patients who have severe clinical illness and report guinea pig exposure.

*Streptococcus equi* subsp. *zooepidemicus* is a facultative pathogen affecting animals and humans. Infections have occurred in horses, pigs, ruminants, guinea pigs, monkeys, cats, and dogs (*1*,*2*). Zoonotic transmission of *S*. *zooepidemicus* is rare and is usually associated with drinking unpasteurized milk or through contact with horses by persons who usually have underlying health conditions (*1*–*3*). Few if any human case-patients with *S*. *zooepidemicus* infection have documented guinea pig exposure even though *S*. *zooepidemicus* infections have been described in guinea pigs since 1907 (*4*). This case report describes 1 probable and 1 confirmed human case of severe *S*. *zooepidemicus* infection and the laboratory methods used to link human and guinea pig isolates.

## Case Reports

An adult man (patient 1) from northern Virginia, USA arrived at a hospital in late February 2013 with influenza-like symptoms, worsening bilateral thigh pain and stiffness, nausea, shivering, fatigue, diarrhea, sweating, and headache. Past medical history included exercise-induced asthma, nephrolithiasis, and slightly elevated liver function test results for “a couple of years,” as stated in the medical record. Initial physical examination of the patient revealed mild scleral icterus, rhabdomyolysis, and rash on his thighs. The patient’s elevated liver function test results were attributed to the rhabdomyolysis. Shortly after being admitted, the patient experienced acute renal failure, sepsis, pneumonia, and bilateral lower extremity edema, the latter of which was thought to be compartment syndrome. Blood cultures performed at the hospital showed group C streptococcal infection. Because of the patient’s worsening condition, he was transferred to a tertiary care center. At the tertiary care center, the patient was treated for septic shock secondary to rhabdomyolysis, placed on a ventilator several times to treat respiratory failure, and underwent bilateral thigh fasciotomy and debridement several times to treat necrotizing fasciitis. Wound cultures identified *S. equi* as the causative agent; a subspecies was not specified, although *zooepidemicus* was likely because it is the only zoonotic subspecies of *S. equi*. After treatment at the tertiary facility for several months, the patient was discharged to a rehabilitation hospital for another month.

An elderly man from central Virginia (patient 2) who was related to patient 1 was admitted to a hospital, 1 week after patient 1 was hospitalized, with nausea, vomiting, chills, difficulty breathing, weakness, abdominal and chest pain, and icterus. The medical history of patient 2 included smoking, oral cancer, myocardial infarction, hypertension, hyperlipidemia, and coronary artery disease. By the second day in the hospital, patient 2 experienced acute hypoxia and respiratory failure; pneumonia in the right lower lobe was diagnosed. He also had hypotension secondary to septic shock and multiple organ failure. Group C *Streptococcus* spp. were identified in blood cultures 2 days after hospitalization. Patient 2 was discharged 18 days after hospitalization and was receiving continuous oxygen.

The local health department for the area in which patient 1 resided was contacted by the tertiary care center where he was treated because of the probability that the *S. equi* infection was caused by guinea pig exposure. Questioning of a female relative of patient 1 (relative 1) indicated that patient 1 recently purchased 4 guinea pigs and that 1 had died shortly after purchase. During the interview with relative 1, it was learned that patient 2 was hospitalized. After another female relative (relative 2) was interviewed, it was learned that patient 2 had cleaned the guinea pigs’ enclosure 2 days before his illness. The Virginia Department of Health (VDH) requested isolates from both patients to be forwarded to the Division of Consolidated Laboratory Services (DCLS), the state public health laboratory. Only an isolate from patient 2 was available and forwarded. VDH representatives also discussed the likelihood of disease in patients 1 and 2 being caused by the guinea pigs with the relatives. Relative 2, who was caring for the guinea pigs at the time, relinquished the 3 remaining guinea pigs which were sent to a Virginia Department of Agriculture and Consumer Services laboratory for euthanasia and microbiological testing.

Using API 20 Strep strips (bioMérieux, Inc., Durham, NC, USA), laboratory personnel identified *S*. *zooepidemicus* in isolates from 5 specimens (1 lymph node, 2 conjunctival swabs, and 2 nasal passage swabs) collected from 2 of the 3 guinea pigs. No apparent lesions were noted in the guinea pigs upon euthanasia and sample collection. Three isolates from the guinea pigs, 1 from each site, were forwarded to DCLS.

At DCLS, patient 2’s isolate was also identified as *S*. *zooepidemicus* by API 20 Strep strips. DCLS staff performed pulsed-field gel electrophoresis (PFGE) testing on all 4 isolates. Briefly, plugs were prepared by incubating washed cells in Tris-EDTA buffer solution (pH 8.0) with 10 μL lysozyme (10 mg/mL) before mixing with 1.5% molecular grade agarose and 0.17 mg/mL proteinase K (Roche, Mannheim, Germany) (*5*). Cells were lysed in extracellular buffer with 1 mg/mL lysozyme and 0.3 mg/mL proteinase K, for 1.5 h at 37°C (*6*). After a MilliQ water wash and 3 Tris-EDTA buffer washes, DNA was digested with 100 U *Sma*I and 100 U *Apa*I both from (New England Biolabs, Inc., Ipswitch, MA, USA) and separated on a 1% agarose gel at 6 V/min, 14°C for 17.5–18 h in 0.5× TBE buffer by using a CHEF Mapper XRS electrophoresis system (Bio-Rad, Hercules, CA, USA). The running parameters were as follows: for *Sma*I: initial pulse, 5.3 sec; final pulse, 34.9 sec; *Apa*I: initial pulse, 2.0 sec; final pulse, 25 sec. The gels were visualized by using ImageLab (BioRad) software with a ChemiDoc XR+ (BioRad). An *Enterococcus faecalis* control strain was used for image normalization during DNA fingerprint pattern analysis with BIONUMERICS 5.1 software (Applied Maths, Austin, TX, USA). The banding patterns were compared by using Dice coefficients with a 1.0%–1.5% band tolerance. PFGE results indicated that all 4 isolates were indistinguishable by comparison with the *Sma*I and *Apa*I enzymes (Figure).

**Figure:**
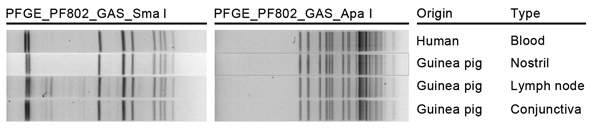
Pulsed-field gel electrophoresis (PFGE) patterns for 4 *Streptococcus equi* subsp. *zooepidemicus* isolates from 1 person and 3 guinea pigs submitted to the Division of Consolidated Laboratory Services, Virginia, USA. Patterns indicate that all 4 isolates were indistinguishable by the *Sma*I and *Apa*I enzymes. Specimen origin and type are indicated.

Multilocus sequence typing was performed as described (http://pubmlst.org/szooepidemicus/) based on the allelic profile of 7 housekeeping gene fragments. The isolates from patient 2 and the 2 guinea pigs shared the same 6-locus profile of 370- to 459-bp housekeeping gene sequences. The 6-locus profile, *arcC27*, *nrdE19*, *spi45*, *tdK1*, *tpi34*, *yqiL46,* was found in each of the 4 isolates rather than a normally obtained 7-locus profile because the *proS* locus did not amplify. This 6-locus profile was unique: its closest match in the *S*. *zooepidemicus* database was ST194 (*arcC27*, *nrdE3*, *proS1*, *spi45*, *tdk1*, *tpi34*, *yqiL46*); both had 5 of the 6 gene-fragment sequences (Table). ST194 is recorded in the *S*. *zooepidemicus* database from 2 human blood isolates recovered during 2001. We subsequently found that ST194 is also shared by ATCC *S*. *zooepidemicus* strain 35246, which was isolated from a diseased pig in China (*7*).

**Table:** Closest species matches to multilocus sequence typing alleles of study *Streptococcus equi* subsp. *zooepidemicus* isolates

Allele*	Length, bp	Closest GenBank overall species match (% identity/overlap)†	Next best GenBank matches (% identity/overlap)†
*arcC27*	437	*S. equi* subsp. *zooepidemicus* strains CY, ATCC35246 (100%/437 bp); H70 (99%/437 bp); MHCS10565 (97%/437 bp)	*S. equi* subsp. *equi* strain 4047 (97%/437 bp)
*nrdE19*	448	*S. equi* subsp. *zooepidemicus* strains CY, ATCC35246, MGCS10565 (99%/448 bp); H70 (97%/448 bp)	*S. equi* subsp. *equi* strain 4047 (94%/448 bp); *S. thermophilus* ASCC1275,JIM8232,ND03, 5 others (80%/447 bp)
*spi45*	459	*S. equi* subsp. *zooepidemicus* strains CY, ATCC35246,4047 (100%/459 bp); MGCS10565 (99%/459 bp); H70 (94%/459 bp)	*S. dysgalactiae* subsp. *equisimilis* strains ATCC12394, 167, AC2713, RE378, GGS124 (72%/447 bp)
*tdk1*	370	*S. equi* subsp. *zooepidemicus* strains CY, 636199–1A, 65843, ATCC35246, H70 (100%/370 bp); 15 others (99%/370 bp)	*S. equi* subsp. *equi* strain 4047 (99%/370bp); *S. equi* subsp. *zooepidemicus* 10 strains (96%–98%/369 bp)
*tpi34*	424	*S. equi* subsp. *zooepidemicus* strains CY, 14580–1A, 44464, ATCC35246 (100%/424bp); 22 others (99%/424 bp); 27 others (95%–97%/424 bp)	*S. equi* subsp. *equi* strain 4047 (97%/424 bp); *S. pyogenes* 20 strains (82%/422 bp)
*yqiL46*	396	*S. equi* subsp. *zooepidemicus* strains CY, ATCC35246 (100%/396 bp); >30 strains (99%/396 bp); >20 strains (95%–98%/396 bp)	*S. equi* subsp. *equi* strain 4047 (97%/424 bp); *S. pyogenes* MGAS6180 (70%/422 bp)

*All 4 isolates shared the identical *arcC27*, *nrdE19*, *spi45*, *tdk1*, *tpi34*, *yqiL46* alleles; sequences provided at http://pubmlst.org/szooepidemicus. †Searches performed at http://blast.ncbi.nlm.nih.gov/Blast.cgi

In addition, the Centers for Disease Control and Prevention laboratory staff sequenced the so-called M-like protein gene (*szp*) of these isolates, using previously described sequencing and amplification primers (*8*). The 1,128-bp structural gene sequence (GenBank accession no. KF722996) was found to be identical for the 4 isolates and closely matched the 1,140-bp M-like gene (*szp*) from the ATCC 35246 strain. The only difference was deletion of 1 of 10 consecutive 4-codon repeats (PKPE, codons 277–280) (*9*).

## Conclusions

*S*. *zooepidemicus* infection should be considered in patients who have purulent wounds or systemic symptoms of infection who have had known contact with guinea pigs or their environment. Likewise, patients whose specimen cultures reveal *S. equi* or further test results show *S*. *zooepidemicus* should be questioned about guinea pig exposure as well as exposure to other animals associated with this pathogen: horses, pigs, ruminants, monkeys, cats, and dogs.

## References

[1] Fulde M, Valentin-Weigard P. Epidemiology and pathogenicity of zoonotic streptococci. Curr Top Microbiol Immunol. 2013;368:49–81. 10.1007/82_2012_277 23192319

[2] Abbott Y, Acke E, Khan S, Muldoon EG, Markey BK, Pinilla M, Zoonotic transmission of *Streptococcus equi* subsp. *zooepidemicus* from a dog to a handler. J Med Microbiol. 2010;59:120–3. 10.1099/jmm.0.012930-0 19745031

[3] Berenguer J, Sampedro I, Cercenado E, Baraia J, Rodríguez-Créixems M, Bouza E. Group-C β-hemolytic streptococcal bacteremia. Diagn Microbiol Infect Dis. 1992;15:151–5. 10.1016/0732-8893(92)90040-Z 1572140

[4] Fraunfelter FC, Schmidt RE, Beattie RJ, Garner FM. Lancefield type C streptococcal infections in strain 2 guinea-pigs. Lab Anim. 1971;5:1–13. 10.1258/002367771781006645 5165756

[5] Benson JA, Ferrieri P. Rapid pulsed-field gel electrophoresis method for group B streptococcus isolates. J Clin Microbiol. 2001;39:3006–8. 10.1128/JCM.39.8.3006-3008.2001 11474035 PMC88282

[6] Bannerman TL, Hancock GA, Tenover FC, Miller JM. Pulsed-field gel electrophoresis as a replacement for bacteriophage typing of *Staphylococcus aureus.* J Clin Microbiol. 1995;33:551–5 . 7751356 10.1128/jcm.33.3.551-555.1995 PMC227989

[7] Ma Z, Geng J, Zhang H, Yu H, Yi L, Lei M, Complete genome sequence of *Streptococcus equi* subsp. *zooepidemicus* strain ATCC 35246. J Bacteriol. 2011;193:5583–4. 10.1128/JB.05700-11 21914890 PMC3187426

[8] Nicholson ML, Ferdinand L, Sampson JS, Benin A, Balter S, Pinto SW, Analysis of immunoreactivity to a *Streptococcus equi* subsp. *zooepidemicus* M-like protein to confirm an outbreak of poststreptococcal glomerulonephritis, and sequences of M-like proteins from isolates obtained from different host species. J Clin Microbiol. 2000;38:4126–30 . 11060079 10.1128/jcm.38.11.4126-4130.2000 PMC87552

[9] Hong-jie F, Fu-yu T, Ying M, Cheng-ping L. Virulence and antigenicity of the szp-gene deleted *Streptococcus equi* ssp. *zooepidemicus* mutant in mice. Vaccine. 2009;27:56–61. 10.1016/j.vaccine.2008.10.037 18983882

